# Role of Goldenberry (Fruits with Husk) Extract in Ameliorating the Architecture and Osmotic Fragility of Red Blood Cells in Obese Rats

**DOI:** 10.1155/2023/8794214

**Published:** 2023-11-27

**Authors:** Sherif A. Abdelmottaleb Moussa, Samir W. Aziz, Noha A. Abd El-Latif, Samir A. E. Bashandy, Marawan A. Elbaset, Sherif M. Afifi, Tuba Esatbeyoglu, Sayed A. El Toumy, Josline Y. Salib

**Affiliations:** ^1^Department of Biochemistry, National Research Centre, P.O. 12622 Cairo, Egypt; ^2^Department of Pharmacology, National Research Centre, P.O. 12622 Cairo, Egypt; ^3^Pharmacognosy Department, Faculty of Pharmacy, University of Sadat City, Sadat City 32897, Egypt; ^4^Department of Food Development and Food Quality, Institute of Food Science and Human Nutrition, Gottfried Wilhelm Leibniz University Hannover, Am Kleinen Felde 30 30167 Hannover, Germany; ^5^Department of Tannins Chemistry, National Research Centre, P.O. 12622 Cairo, Egypt

## Abstract

Goldenberry (GB) is a promising fruit that can be a constituent in many possible nourishments. No notifications were obtained regarding the impact of exposure to goldenberry extract in the viewpoint of blood rheological properties as well as erythrocyte osmotic fragility of red blood cells (RBCs) in obese rats. A substantial reduction in plasma triglyceride, total cholesterol, and LDL, with a considerable increment in HDL levels relative to the obese group (*p* ≤ 0.05), was observed in rats receiving low and high doses of GB, accompanied by restoration of SOD activity and GSH levels. Rheological parameters of rats' blood have been studied over a wide range of shear rates (225–1875 s^–1^). A significant decrease in blood viscosity in rats who received low and high doses of GB extract was compatible with every shear rate compared to the control group. The shear stress values of the obese rats reduced appreciably (*p* ≤ 0.05) in all values of shear rate (from 75 to 500 s^−1^) proportional to the control group, while in the groups that received low and high doses of GB extract, shear stress was restored to the control values. Finally, administration of GB extract significantly decreased yield stress and indices of whole blood aggregation, with an extremely substantial increment in flow rate, in rats given low or high doses of GB compared to obese ones. The result also showed a decrease in both the average raised osmotic fragility and the hemolysis rate in rats after supplementation with low and high doses of GB extract.

## 1. Introduction

Obesity is a morbid state where the surplus of fat is stocked to the limit, which may harm health and shorten life [1]. Obesity is more associated with free radical stress, which generates a vast area of inflammation and metabolic disorders [2, 3]. It neatly correlates to accumulated body injuries due to unbalanced free radicals by antioxidant components [4].

Free radicals possess a passive effect on the viability of the cell due to the harm of the membrane induced by oxidative lipids, markers of oxidative injury to interactive oxygen species (ROS), and pointers for the oxidation of protein [5].

Blood rheological disorders regarded as obesity are considered a hazard factor for sundry comorbid diseases since they play a considerable part in microcirculation blood flow [6]. Hemorheological fluctuations in obesity, such as deviations in the blood's rheological manner, increment in blood viscosity, and red cell aggregation, have been recorded by several authors [7, 8].

Blood is an unparalleled fluid. It displays non-Newtonian characters, so the viscosity of blood relies on the shear rate. The prime factors of the viscosity of whole blood are hematocrit, plasma viscosity, aggregation, and distortion of red blood cells under conditions of low and high shear rates [9, 10].

The rheological blood properties broadly rely on the properties of RBCs [11]. Another substantial characteristic of RBCs is their aggregation. It tends to compose stacked structures, popularly known as “rouleaux,” which affects the rheological properties of blood as it seems to increase blood viscosity, thus decelerating its flow [12, 13].

Osmotic fragility is most used to interpret the mechanisms of the impact of various factors on the osmotic properties of erythrocyte membranes. The osmotic fragility test is valuable in diagnosing some blood diseases [14]. Low osmotic resistance may be due to intravascular hemolysis, which reduces the RBC lifespan [15]. The curve of osmotic fragility's red cells not only manifests average membrane and cytoplasmic properties but may also contribute to reporting the distribution of those properties in the sample [16].

The current study sheds light on the prominent effect of goldenberry extract as a safe, edible natural product for improving the rheological properties of blood as well as the osmotic fragility of RBCs; this is due to its luminal effect as a scavenger of free radicals, which leads to the reduction of the lipid peroxidation.

## 2. Materials and Methods

### 2.1. Plant Material and Reagents

*Physalis peruviana* fruits with husk were gathered from the store in March 2021 in Cairo, Egypt. The Herbarium of the Botany Department, Faculty of Science, Cairo University, identified the plant. In Cairo, Egypt's National Research Center Herbarium, voucher specimens were placed. The Folin–Ciocalteu reagent is prepared by mixing sodium tungstate and phosphomolybdic acid in phosphoric acid, and the AlCl_3_ reagent was obtained from Sigma (St. Louis, MO) without further modifications.

### 2.2. Preparation of Extract

About 5 kg of fresh fruits with their husk was washed by running a water tap; a blender was used to grind the lyophilized fruits with husk, followed by soaking in 70% methanol, heating at 40°C, and then filtering in a sterile flask. Replication of this process three times was performed at 40°C. After that, the extract was collected, filtered, and concentrated using a rotary evaporator Heidolph (Rothenburg ob der Tauber, Germany) at 45°C under vacuum; the crude extract was lyophilized to obtain a dry residue (60 g) and then restored in a freezer at -4°C until usage.

### 2.3. Determination of Total Phenolic and Total Flavonoid Content

The entire phenolic component (TPC) of the extract was determined as per the guidance of [17] and expressed as gallic acid equivalent (GAE)/g DW extract of berry. Besides, the total flavonoid component (TFC) was estimated according to [18–20] and expressed as the rutin equivalent (RE)/g of dried berry extract [21, 22].

### 2.4. Animal Care and Treatments

Thirty-two adult Wistar female rats of weight 155-175 g (Animal House Colony of National Research Centre, Egypt) were housed in ambient conditions with similar cycles of dark and light and *ad libitum* to food and water for one week. Then, 8 rats were maintained in the same condition for the end of the study. At the same time, the 24 rats were fed a high-fat diet for 56 days, as ascribed by El-Seidy et al. [23], under the ethical permission of 19161 by the Research Ethics Committee of the National Research Centre.

The rodents were divided evenly (eight to each group) into four categories.

Rats in the control group were provided with a regular free chow meal and a vehicle for three months to serve as a comparison. Three months of a high-fat diet (HFD) [24] and 25% sugar in tap water were used to induce obesity in the control rats.

The other two groups of GB extract at a modest dose of 200 or 400 mg DW/kg b.w. (body weight) [25, 26] were given orally to obese rats for 2 months.

### 2.5. Samples Collection

Under diethyl ether local anesthetic, the saphenous vein blood was collected in heparinized tubes after three months. For isolation, plasma was centrifuged at 3,000 g for 15 min. The isolated aliquot was kept in the fridge until analyses. Anesthetized animals were sacrificed, and their hearts and aortas were excised for histopathology.

### 2.6. Lipid Profile Test

The profile of lipids was estimated by the calorimetric method utilizing Salucea Company, Haansberg, Netherlands, kits according to the manufacturer's protocol.

### 2.7. Plasma Antioxidant and Oxidative Stress Markers

Thiobarbituric acid-malondialdehyde adduct (TBARS), superoxide dismutase (SOD), and reduced glutathione (GSH) were determined in plasma and heart tissues and evaluated calorimetrically using Biodiagnostic kits (Cairo, Egypt) [27].

### 2.8. Rheological Measurements

Blood viscosity (cp), shear stress (Dyne/cm^2^), and Torque (%) were determined at shear rates of 225-1875s^−1^ and 75-900 s^−1^ for whole blood rats. Brookfield LVDV-III programmable cone-plate rheometer (Brookfield Engineering Laboratory, Incorporation, Middleboro, USA) was applied for measuring rheological parameters. The values of viscosity between 1.5 to 30,000 m Pas were measured with reproducibility of ±0.2%. A cone and plate sensor of a diameter of 2.4 cm and angle of 0.8 and an SC-40 spindle, which has high precision at torques of 10% to 100%, were applied. A sensor temperature has been utilized to regulate the temperature accurately inside the sample chamber through the measurements and then taken in a computer-controlled water bath at 37°C. An aliquot of each sample (whole blood) 0.5 mL was put into the rheometer sample chamber. Samples were then immersed and spun by the spindle at constantly increasing speeds (20-180 rpm) for 20 min [28].

### 2.9. Erythrocyte Osmotic Fragility

Measurement of the erythrocyte osmotic fragility of red blood cells was performed as stated in [29].

### 2.10. Statistical Analysis

Values were given as the mean ± standard error. The significance of the differences between the groups' variances was examined using analysis of variance (ANOVA) and then the LSD comparison test, which was calculated using SPSS software, version 21. *p* ≤ 0.05 was the statistical significance threshold.

## 3. Results

### 3.1. Total Phenolic and Total Flavonoid Content

**Figure 1:**
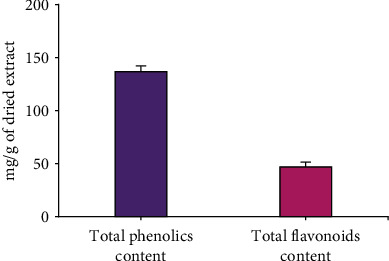
Total phenolic and flavonoid contents in the fruit of *Physalis peruviana* with husk extract. Bars represent mean ± SEM, *n* = 3.

The total phenolic (TPC) and total flavonoid contents (TFC) of GB fruits with husk extract were evaluated in (Figure 1). TPC was determined as gallic acid equivalent (GAE)/g DW extract of berry and TFC as rutin equivalent (RE)/g of dried berry extract.

### 3.2. Effect of Goldenberry Supplementation on Lipid Profile of Obese Rats

A marked increase in serum triglycerides (TG), total cholesterol, and LDL, associated with a considerable decrease in levels of serum HDL regarding group control (*p* ≤ 0.05), was observed (Table 1). Thus, there was an impairment in the levels of profile lipids in the obese rats. In contrast, there was a marked decrement in serum triglyceride, total cholesterol, and LDL, with a considerable increase in HDL in rats that received low and high doses of GB levels in contrast to the obese rats (*p* ≤ 0.05). Moreover, in the group of rats given 400 mg/kg GB, the levels of triglycerides, total cholesterol, LDL, and HDL were close to those in the control group.

### 3.3. Influence of Supplementation of Goldenberry on Parameters of Plasma Antioxidants and Oxidative Stress of Obese Rats

Table 2 illustrates the antioxidant indices and enzymes in the plasma of all groups. Plasma TBARS content was applied to discriminate the reactive oxygen species versus control rats since animals over HF-dependent animals had higher plasma lipid peroxidation (*p* ≤ 0.05). GB supplementation was spotted to improve lipid peroxidation (*p* ≤ 0.05) compared to obese rats. GSH and SOD are antioxidants inherent in revolving plasma that assists in lessening oxidative strain, as shown in Table 2 (*p* ≤ 0.05). In this search, the activity of SOD was extremely reduced (*p* ≤ 0.05) in the plasma of obese rats fed an HF diet regarding control rats, while GSH was decreased spectacularly in the plasma of obese rats in contrast to the control group. Supplementation of GB extract in both the low- and high-dose groups was observed to significantly restore the activity of SOD and GSH levels (*p* ≤ 0.05) compared to the obese rats.

### 3.4. Effect of Goldenberry Supplementation on Rheological Properties of Blood of Obese Rats

**Figure 2:**
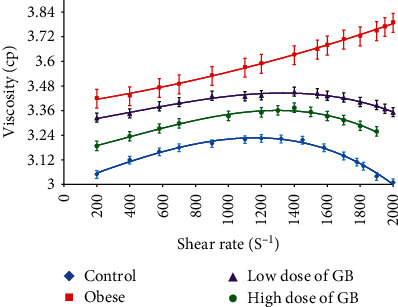
The viscosity of blood in a broad range of shear rates (s^−1^) for obese, obese + 200 mg/kg GB extract, and obese + 400 mg/kg GB extract groups versus the control group (Mean ± SEM).

The relevance among viscosity (cp) and shear rate (s^−1^) was measured for all rats' blood groups with overbroad shear rates ranging from 225 to 1875 s^−1^ and 37°C fixed temperature as shown in (Figure 2); a considerable increment was mentioned in the obese versus the control group. Conversely, the blood viscosity of rats that received either low or high doses of GB conformable to each shear rate was significantly reduced in contrast to the normal; the viscosity values approached the control values for all shear rate values. In addition, the region of low shear can be known for its consistency index (low shear viscosity) and flow index.

**Figure 3:**
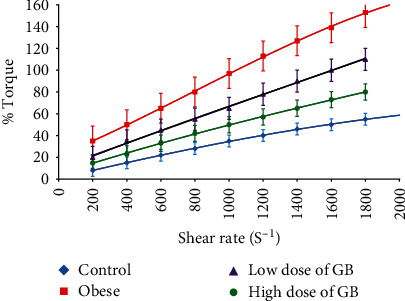
The blood % Torque in a broad range of shear rates for obese, obese + 200 mg/kg GB extract, and obese + 400 mg/kg GB extract groups versus the control group (Mean ± SEM).

The blood % Torque and shear stress (Dyne/cm^2^) (Figure 3) were measured at shear rates for a broad range of (225-1875s^−1^) at 37°C for blood rat groups. The relation between % Torque and shear rate demonstrated a linear behavior for all rat groups. To obtain values of the actual viscosity from the Brookfield rheometer, it was demonstrated that the value of the upper shear rate was approximately 950 s^−1^ at the high value of % Torque, but the minimized value of the shear rate was about 85 s^−1^ at the lowest % Torque value.

**Figure 4:**
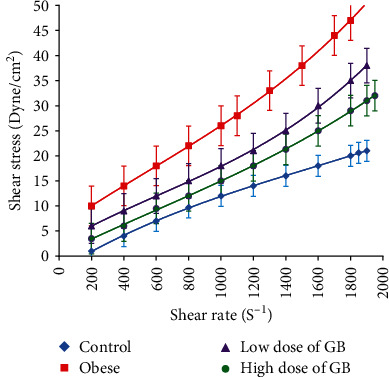
Blood shear stress (Dyne/cm^2^) in a broad range of shear rates for obese, obese + 200 mg/kg GB extract, and obese + 400 mg/kg GB extract groups versus the control group (Mean ± SEM).

The relevance between shear stress and shear rate values for all groups was linearly related, as shown in Figure 4. It was found that the values of the shear stress of the obese rats markedly lowered (*p* ≤ 0.05) at all values of shear rate from 75 to 500 s^−1^ versus the control group; while the groups were subjected to low and high doses of GB extract, shear stress returned towards their control values at all values of shear rate.

Table 3 shows a highly remarkable decrease in blood viscosity, consistency index, and the value of the yield stress for low and high doses of GB extract groups versus the obese rats, accompanied by a highly significant increment in the flow index. In contrast, a considerable increase in the obesity group was spotted in all subjects relative to the control group.

### 3.5. Effect of Goldenberry Supplementation on Erythrocyte Osmotic Fragility of Obese Rats

**Figure 5:**
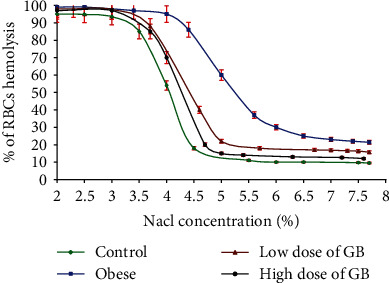
Curves of red blood cells osmotic fragility for obese, obese + 200 mg/kg GB extract, and obese + 400 mg/kg GB extract groups versus the control group (Mean ± SEM).

Meanwhile, the hemolysis dispersion (*S*) illustrated a severe decrease (Table 4). The results acquired from the obesity curve revealed a higher hemolysis rate (*P*) with an accompanying shift of the center of the peaks (*C*) to higher sodium chloride concentration values (Figure 5).

**Figure 6:**
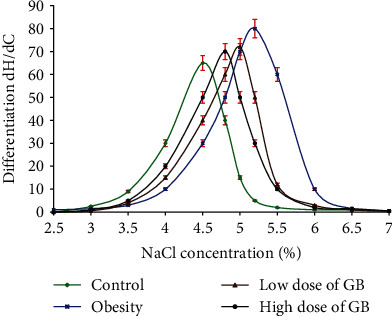
Gaussian curves (hemolysis rate versus NaCl concentration) of red blood cells for obese, obese + 200 mg/kg GB extract, and obese + 400 mg/kg GB dried extract groups versus the control group (Mean ± SEM).

Both groups bared to a low or 400 mg/kg GB showed a lessening action of free radicals on the safety structure of erythrocytes (Figures 5 and 6 and Table 4).

## 4. Discussion

Obesity is a broadly complicated disease, which accounts for more than a third of the population today [30, 31]. If worldly tendencies keep, by 2030, 38% of adults in the world will be obese and other 20% obese [32]. Obesity considerably raises the risk of developing chronic disease morbidity [33]. Thus, the financial and psychosocial value of obesity, when incorporated with these complications, is staggering.

The results of the four groups' lipid profiles (cholesterol, HDL, LDL, and triglyceride) showed an improvement in lipid profiles after GB extract, primarily at a high dose. The results acquired pointed out that cholesterol, TG, and LDL levels were markedly increased in the obese rats concomitantly with a significant decrement in the HDL levels compared to the control group. Our results were proportionate with former studies [34, 35].

Increment lipid peroxidation is accompanied by a decrement in levels of GSH and SOD, pointing out that oxidative stress results from free radicals in obesity. It is worthy to mention that GB improved antioxidant properties of obese rats by enhancement of antioxidant enzymes which was proportionate to the results of [36].

The antioxidant role of GB may be due to the entity of flavonoid and phenolic compounds present in GB (Table 1). Flavonoid compounds are recognized as possible antioxidants because of their power as scavengers against free and species of active oxygen, such as singlet oxygen, superoxide anion radicals, and hydroxyl radicals [37, 38]. TBARS is extremely prevalent in lipid peroxidation products, and its high levels may be responsible for the risk of damage by the peroxidation of lipids in red blood cells [39]. The selected GB extract significantly reduced TBARS levels, and the levels indicate its antiperoxidative effect.

Another motivating finding of our work was that treatment with GB extract attenuated the whole blood rheological properties study and varied the viscosity together with the shear rate. Since blood is a non-Newtonian suspension, its fluidity cannot be determined by one value of viscosity. Rheometer allows viscosity to be measured through a range of shear rates, resulting in a flow curve for a blood sample. Low shear viscosity fundamentally builds on the erythrocyte aggregation, while high shear viscosity relies on the erythrocytes' deformation. This deformation is accountable for the low-value viscosity at higher shear rates [40]. Whole-blood viscosity builds on the geometry of the blood's physiological constituents. Obesity results from major components of metabolic syndrome without influencing blood vessels and microcirculation. Metabolic syndrome is a case of oxidative stress and systemic inflammation [41]. Morphological variations in erythrocytes may be due to reduced deformability of erythrocytes, oxidative stress, and systemic inflammation.

In obese rats, blood viscosity increases as the hematocrit level rises. Decreased deformation, as well as the increase in shear stress, may be accountable for the rise in the viscosity of blood. Furthermore, a decrease in the deformation of RBCs is associated with increased accumulation of RBCs and increased disaggregation of RBCs' shear rate [42]. The functional damage to RBCs may be clarified by the persistent oxidative stress which affects obese rats. High TBARS and low GSH concentrations reflect reduced antioxidant protection.

Reports have shown that many changes in the properties of hemorheology may occur due to the produced free radicals, such as lipid peroxidation. Lipid peroxidation may result from the decrease in the red cells' deformability and the increase in aggregation [43].

Our findings indicated that obese rats subjected to GB developed a decrement in the rheological alterations compared to untreated obese rats. The amelioration in rheological changes can be referred to as the direct effect of GB as an antioxidant elicited by lowering plasma TBARS and/or enhancing plasma GSH and SOD [44–46]. Ascorbic acid and phenolics found in GB fruit may participate in the high capacity of antioxidant level and high free radicals scavenging agent. Flavonoids and phenolics from golden berries have higher radical scavenging and antilipid peroxidation activity [47].

The data gained in this study pointed to a marked considerable reduction in the blood viscosity, consistency index, and yield stress value accompanied by a highly considerable increase in blood flow rate in rats subjected to low or high doses of GB extract. These changes in the conformational structure may be due to GB extract containing high levels of total phenolics content, total flavonoids content as in Table 1, unsaturated fatty acids, and polyphenols, which are excellent reactive oxygen scavengers and provide promising chelating effects for Fe overload [48]. We hypothesized that the phenolics and flavonoids present in GB extract interacted with Fe to enhance Fe excretion rates and had a ferric-reducing power test, improving the iron deficiency anemia associated with obesity [49].

## 5. Conclusions

The utmost goal of ongoing studies is the osmotic fragility of erythrocytes which is considered an indirect method for estimating oxidative stress as a valuable way for a human health screening with morbid troubles. This study highlights the potential health benefits of goldenberry (GB) extract in obese rats, specifically concerning blood rheological properties and erythrocyte osmotic fragility. The findings demonstrate that administering low and high doses of GB extract resulted in significant improvements in lipid profiles, with reductions in plasma triglyceride, total cholesterol, and LDL levels and an increase in HDL levels. Additionally, the study observed restored antioxidant enzyme activity and reduced blood viscosity in rats receiving GB extract, indicating enhanced blood flow and potential cardiovascular benefits. Furthermore, the supplementation of GB extract decreased yield stress, improved indices of whole blood aggregation, and reduced erythrocyte fragility. These findings suggest that GB extract has potential therapeutic applications in managing obesity-related complications and improving blood rheology.

Moreover, this study contributes to obtaining information regarding ion transport across the cell membrane, mechanical properties, and membrane structure, which are very important for cell-to-cell communication and metabolic activities of RBCs. Its remarkable role is hypocholesterolaemia, as cholesterol is an essential compound in cell membranes as it organizes membranes over various physiological temperatures. Further research is warranted to elucidate the underlying mechanisms and validate these results in human studies.

## Figures and Tables

**Table 1:** Lipid profile of four experimental groups (control, obesity, and low- and high-dose goldenberry).

Lipid profile indices	Groups			
	Control	Obese	Obese + 200 mg/kg GB	Obese + 400 mg/kg GB
Triglyceride (mg/dl)	48.1 ± 2.98	178.7 ± 11.18^∗^	57.4 ± 5.31^@^	45.6 ± 6.43^@^
Total cholesterol(mg/dl)	55.4 ± 1.23	101.1 ± 3.16^∗^	64.1 ± 0.646^@^	60.2 ± 1.56^@^
HDL (mg/dl)	45.7 ± 1.26	41.7 ± 0.881^∗^	40.2 ± 6.73^@^	44.8 ± 3.16^@^
LDL (mg/dl)	220.1 ± 3.54	264.3 ± 5.75^∗^	209.0 ± 3.27^∗^^@^	211.4 ± 3.90^@^

Each reading represents the mean ± SE (^∗^ versus control rats and @ versus obese rats) at *p* ≤ 0.05.

**Table 2:** The plasma's antioxidant and oxidative stress parameters for obese and obese + 200 or 400 mg/kg GB extract groups, relative to control.

Antioxidant indices	Groups			
	Control	Obese	Obese + 200 mg/kg GB	Obese + 400 mg/kg GB
GSH (mg/dl)	24.0 ± 1.99	8.30 ± 0.81^∗^	21.6 ± 1.11^@^	22.5 ± 0.75^@^
SOD (U/ml)	54.4 ± 6.11	18.2 ± 1.5^∗^	27.0 ± 1.9^@^	40.8 ± 2.1^@^
TBARS (nmol/ml)	0.790 ± 0.07	8.46 ± 0.81^∗^	1.19 ± 0.14^@^	0.800 ± 0.05^@^

Each reading represents the mean ± SEM (^∗^ versus control rats and @ versus obese rats) at *p* ≤ 0.05.

**Table 3:** Viscosity, yield stress, consistency, flow, and aggregation indices for obese, obese + 200 mg/kg GB extract, and obese + 400 mg/kg GB extract groups versus the control group.

Rheology indices	Groups			
	Control	Obese	Obese + 200 mg/kg GB	Obese + 400 mg/kg GB
Viscosity (cp)	3.12 ± 0.14	8.35 ± 0.27^∗^	6.98 ± 0.17^@^	4.33 ± 0.15^@^
Yield stress (Dyne/cm^2^)	0.401 ± 0.05	0.932 ± 0.09^∗^	0.711 ± 0.08^@^	0.536 ± 0.065^@^
Consistency	20.7 ± 1.96	26.3 ± 2.87^∗^	24.0 ± 1.72^@^	21.1 ± 1.66^@^
Flow index	0.673 ± 0.02	0.987 ± 0.06^∗^	0.754 ± 0.04^@^	0.701 ± 0.03^∗^^@^
Aggregation index	1.56 ± 0.05	3.34 ± 0.12^∗^	2.98 ± 0.081^@^	2.01 ± 0.071^∗^^@^

Each reading represents the mean ± SEM (^∗^ versus the control group and @ versus obese rats) at *p* ≤ 0.05.

**Table 4:** H50 center, width (*S*), and height (*P*) of Gaussian peaks red blood cells for obese, obese + 200 mg/kg GB extract, and obese + 400 mg/kg GB extract groups versus the control group.

Group	Average osmotic fragility H_50_ (g/l)	Dispersion of hemolysis (*S*) (g/l)	Area (*A*) (mm^2^)	Height (*P*) %
Control	4.45 ± 0.006	0.990 ± 0.006	85.1 ± 0.573	70.24
Obese	5.82 ± 0.002^∗^	0.850 ± 0.005^∗^	83.9 ± 0.532^∗^	83.45
Obese + 200 mg/kg GB	4.93 ± 0.004^∗^^@^	0.910 ± 0.006^∗^^@^	84.4 ± 0.543^∗^^@^	68.03
Obese + 400 mg/kg GB	4.35 ± 0.000^∗^^@^	0.960 ± 0.006^∗^^@^	84.9 ± 0.56^∗^^@^	70.01

Each reading represents the mean ± SE M (^∗^ versus the control group and @ versus obese rats) at *p* ≤ 0.05.

## Data Availability

All data is within the manuscript. Further details can be requested from the first author.
